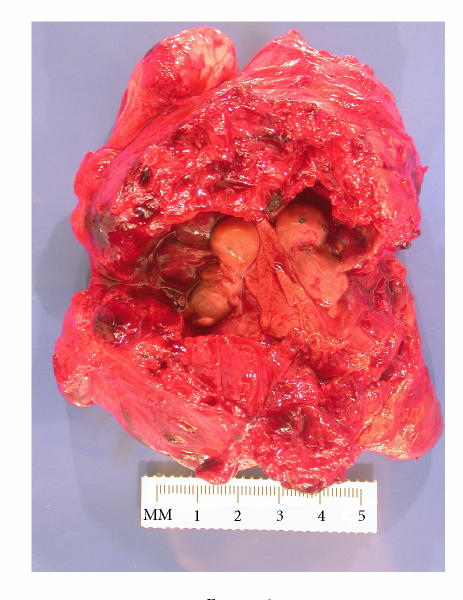# Unilateral Twin Ectopic Pregnancy in a Patient With a History of Multiple Sexually Transmitted Infections

**DOI:** 10.1155/IDOG/2006/10306

**Published:** 2006-06-14

**Authors:** Charles J. Rolle, Clifford Y. Wai, Roger Bawdon, Rigoberto Santos-Ramos, Barbara Hoffman

**Affiliations:** Department of Obstetrics and Gynecology, University of Texas Southwestern Medical Center, Dallas, TX 75390, USA

## Abstract

*Background*. The incidence of unilateral twin ectopic pregnancy is a rare condition. Several factors increase the risk of ectopic pregnancy, the most important of which is pelvic inflammatory disease, followed by operative trauma, congenital anomalies, tumors, and adhesions resulting in anatomically distorted fallopian tubes. We present a case of a woman with a history of four confirmed sexually transmitted infections (STIs) including *Chlamydia trachomatis, Neisseria gonorrhoeae*, herpes simplex virus 2, and *Treponema pallidum*. The case illustrates the potential impact of sexually transmitted infections (STIs) on the risk of a twin ectopic pregnancy. *Case*. A 24-year-old primigravida, presented with an unknown last menstrual period, lower abdominal pain, watery vaginal discharge, and vaginal spotting. During this hospitalization, serum β-HCG testing was 263 mIU/mL and transvaginal ultrasonographic examination suggested a nonviable unilateral twin ectopic pregnancy. At exploratory laparotomy, a 10 cm mass involving the right fallopian tube and ovary was excised. Pathological evaluation of the specimen identified a monochorionic, diamnionic twin ectopic pregnancy within the fallopian tube. *Conclusions* Patients with a history of multiple (STIs) are known to be at risk for the development of chronic pelvic infection and postinflammatory scarring. The resulting distortion of the normal tubal anatomy leads to an increased risk of an uncommon presentation of ectopic pregnancy.

## INTRODUCTION

Patients with a history of sexually transmitted infections (STIs) are at increased risk for the development of ectopic pregnancies because of postinflammatory fallopian tube scarring and deformity. Unilateral twin ectopic pregnancy is a rare condition, first described in 1891 by De Ott [1]. Live twin ectopic pregnancies are thought to occur at a frequency of 1/125 000 [2]. Unilateral twin ectopic gestation is thought to occur in approximately 1 in 200 ectopic gestations [3]. One would therefore calculate a frequency of 1 in 20 000 pregnancies with a 1% ectopic rate in the United States. Simultaneous bilateral tubal pregnancy is the rarest form of double-ovum twin pregnancy.

## CASE

A 24-year-old primigravida with an unknown last menstrual period presented to the emergency room with a 3-month history of lower abdominal and lower back pain that radiated into her right thigh. The pain occurred two to three times daily, lasting 5 minutes with each episode and worsening during the few days prior to presentation. During the three previous months the patient described irregular vaginal bleeding and watery vaginal discharge. She was evaluated two months previously at another hospital and was diagnosed as having a “cyst.” She was discharged with a narcotic prescription that she noted had improved her pain, but did not relieve it.

Her medical history revealed no prior surgeries or any history of assisted reproduction. Coitarche began at 14 years of age and she noted a minimum of 15 previous sexual partners. Hospital medical records listed positive toxicology screen results for illegal drugs on multiple emergency room visits. During the previous 6 years, culture and serology results documented four prior separate infections with *Chlamydia trachomatis, Neisseria gonorrhoeae*, herpes simplex virus 2, and *Treponema pallidum*, respectively. The patient reported that she completed treatment for all four infections. Current testing for *C trachomatis* and *N gonorrhoeae* by DNA probe was negative for both species. Serum testing for rapid plasma reagin (RPR) was also negative. The patient did not demonstrate any clinical sequelae of herpes simplex virus and thus testing for this STI was not performed on the current admission.

On physical exam, the patient was afebrile, and her vital signs were stable. Abdominal palpation elicited tenderness in the right lower quadrant. Pelvic examination revealed blood in the vagina, a closed cervical os, uterine tenderness, and a palpable right adnexal mass. Rectovaginal exam concurred with the pelvic exam and stool guaiac testing for occult blood was negative.

**Figure 1:**
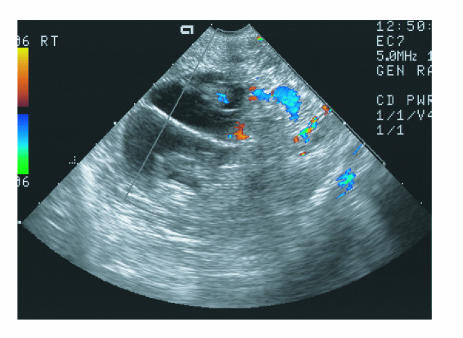


Diagnostic testing showed a quantitative serum β-HCG titer of 263 mIU/mL, a hematocrit of 38%, and a serum progesterone level of 2.2 ng/mL. Additionally, ultrasonographic examination displayed a 10 × 10 × 8 cm right adnexal mass containing a nonviable twin pregnancy with each fetal crown-rump length corresponding to a gestational age of 9.3 weeks. A separating linear echo was seen in the mass between the two fetuses (Figure 1).

An exploratory laparotomy revealed a hemoperitoneum containing 300 mL and a 10 cm right-sided pelvic mass incorporating the right fallopian tube and ovary. Small intestine and the appendix were adherent by fibrous adhesions to the mass. The contralateral ovary and fallopian tube had numerous adhesions distorting their normal anatomy. A right salpingoophorectomy, an appendectomy, and an extensive lysis of adhesions were performed. The patient's postoperative course was uncomplicated.

Pathological evaluation of the surgical specimen showed a twin pregnancy within the fallopian tube (Figure 2). During more detailed histopathologic examination, the finding of a dividing membrane established the pregnancy to be monochorionic diamnionic, and measurement of the twin fetuses estimated their gestational age at 7 weeks.

## DISCUSSION

Several factors are thought to increase the risk for ectopic pregnancy. Pelvic inflammatory disease is associated with the greatest increase in risk, but other associated factors include operative trauma, congenital anomalies, tumors, adhesions, and advancing maternal age [4].

Arey in 1923 suggested that anything that interferes with the passage of the ovum through the tube increases the risk of an abnormal placental implantation site [5]. Additionally, studies suggest a delay in ovum transport and in implantation also increase the risk of monozygotic twinning [6]. Minor trauma to the blastocyst during assisted reproductive technology may also lead to an increased incidence of monozygotic twinning [7]. Correspondingly, the majority of twin ectopic pregnancies have been monozygotic. As of 1990, 95% of twin ectopic pregnancies were reported as monozygotic [8].

Despite these potential sources for ectopic twin gestation, the reported incidence is low. Goker in 2001 reported only the one hundred and first case of unilateral twin ectopic pregnancy, and the sixth to be diagnosed preoperatively [8]. The current case is the eighth in the literature to be diagnosed preoperatively. One reason for the low incidence may stem from the fact that fetal wastage in monozygotic twins is high and malformations are common [9].

In the current case, the patient's prior STIs may have had a direct causal link to her ectopic twin gestation. Current studies show that *C trachomatis* and *N gonorrhoeae* interfere with the function of the ciliated cells of the tubal mucosa, thereby slowing ovum transport and increasing the risk for tubal implantation. Gérard reported that 7 of 10 tubes with ectopic pregnancies were found to have *C trachomatis* [10]. In addition to these findings, it has been demonstrated with laparoscopy that one episode of PID resulted in a 12.8% incidence of bilateral tubal occlusion, two episodes with a 35% incidence, and three episodes with a 75% incidence of bilateral tubal occlusion.

## CONCLUSIONS

This case illustrates one of the long-term consequences of STIs with respect to reproductive function. Patients with a history of multiple STIs are known to be at risk for development of chronic pelvic infection and its associated fallopian tube scarring. The tubal anatomy distortion that ensues potentially increases the risk for a presentation of ectopic pregnancy that is considered uncommon.

## Figures and Tables

**Figure 2:**